# Cellular and Molecular Mechanisms Underpinning Macrophage Activation during Remyelination

**DOI:** 10.3389/fcell.2016.00060

**Published:** 2016-06-21

**Authors:** Amy F. Lloyd, Veronique E. Miron

**Affiliations:** MRC Centre for Reproductive Health, Queen's Medical Research Institute, University of EdinburghEdinburgh, UK

**Keywords:** remyelination, microglia, macrophages, regeneration, inflammation, plasticity, multiple sclerosis, myelin

## Abstract

Remyelination is an example of central nervous system (CNS) regeneration, whereby myelin is restored around demyelinated axons, re-establishing saltatory conduction and trophic/metabolic support. In progressive multiple sclerosis, remyelination is limited or fails altogether which is considered to contribute to axonal damage/loss and consequent disability. Macrophages have critical roles in both CNS damage and regeneration, such as remyelination. This diverse range in functions reflects the ability of macrophages to acquire tissue microenvironment-specific activation states. This activation is dynamically regulated during efficient regeneration, with a switch from pro-inflammatory to inflammation-resolution/pro-regenerative phenotypes. Although, some molecules and pathways have been implicated in the dynamic activation of macrophages, such as NFκB, the cellular and molecular mechanisms underpinning plasticity of macrophage activation are unclear. Identifying mechanisms regulating macrophage activation to pro-regenerative phenotypes may lead to novel therapeutic strategies to promote remyelination in multiple sclerosis.

## Introduction

A prime example of regeneration in the central nervous system (CNS) is remyelination, whereby following demyelination, myelin is regenerated around axons allowing recovery of saltatory electrical impulse conduction and metabolic/trophic support. This involves recruitment and proliferation of oligodendrocyte progenitor cells (OPCs) which subsequently need to survive and differentiate into mature myelinating oligodendrocytes. Remyelination is limited or fails altogether in the progressive phase of the autoimmune disease multiple sclerosis (MS), which is considered to contribute to the axonal damage and loss that correlates to sensory, motor and cognitive decline. Given that there are no approved therapies to drive remyelination in MS, identifying the cellular and molecular drivers of remyelination is key in the development of novel therapeutics.

One of these cellular components includes cells of the innate immune system, such as macrophages derived from CNS-endogenous microglia and monocyte-derived macrophages. Macrophages are the first line of defense against infection and injury, involved in the phagocytosis of apoptotic cells/debris, antigen presentation, secretion of growth/neurotrophic factors/cytokines, and recruitment of other immune cell subsets. Although, initially implicated in tissue damage, recent studies have expanded their roles to include tissue regeneration such as remyelination, relating to their ability to phagocytose myelin debris and to secrete regenerative molecules. Indeed, minocycline-induced inhibition of microglia activation significantly impairs remyelination (Li et al., 2005). The diversity of macrophage function reflects acquisition of a diverse spectrum of activation states in response to stimuli in their microenvironment. The plasticity of this activation has been demonstrated using models of demyelination showing that microglia adopt different phenotypes during distinct stages of myelin damage and regeneration (Olah et al., 2012; Voß et al., 2012; Miron et al., 2013). Importantly, the phenotypic profile of microglia during remyelination has been correlated with functional changes, including phagocytosis of myelin debris and apoptotic cells (Olah et al., 2012) and regulation of oligodendrocyte progenitor cell responses (Miron et al., 2013). These findings indicate that timed coordination of particular macrophage phenotypes and functions are essential for efficient remyelination, although the cues dictating their activation and phenotypic switching remain poorly understood. Gaining an understanding of these processes is imperative in order to develop more effective therapeutic interventions for neurodegenerative diseases like MS. This review will address the roles of functionally distinct macrophage phenotypes during regeneration (e.g., remyelination), the plasticity of this activation and the molecular mechanisms involved.

## Activation and functions of microglia and macrophages during CNS regeneration

The extensive functions adopted by macrophages reflects their response to their microenvironment, defined by interactions with other cells, secreted products (such as cytokines), and signals of injury (damage associated molecular patterns; DAMPS) or infection (pathogen associated molecular patterns; PAMPS). These microenvironment-specific functional macrophage responses have been associated with marker expression changes, termed “activation” phenotypes. Whereas earlier studies categorized these activation phenotypes broadly into 2 categories, with “M1” used as an umbrella term for pro-inflammatory activation and “M2” used to describe anti-inflammatory/immune-regulatory/wound-healing phenotypes, recent studies have demonstrated that use of this terminology can be misleading as various stimuli used to induce one of these activation states can cause distinct gene expression changes (Xue et al., 2014). Therefore, in order to avoid assumptions of gene expression or function, and to achieve reproducible findings in the macrophage field, Murray et al. (2014) proposed to define macrophage activation by stimuli used, experimental conditions, and a combination of markers. Therefore, we have adopted this method to describe studies investigating microglia and macrophage activation in CNS regeneration in this review.

Although, *in vitro* stimulation of macrophages may not recapitulate the complexity of the CNS microenvironment, its minimalistic approach has given useful insight into activation state-associated gene expression and biochemical/functional responses (Edwards et al., 2006). For example, *in vitro* activation using interferon-gamma (IFNγ) and bacterial peptide lipopolysaccharide (LPS), IFNγ and Fc receptor (FcγR) stimulation, or interleukin-4 (IL-4) produce unique gene, transcript and protein expression patterns, such that only macrophages exposed to IFNγ express inducible nitric oxide synthase (iNOS), whilst IL-4-treated macrophages are the only group to express arginase-1 (Edwards et al., 2006). *In vitro* experiments have identified potential molecular mechanisms regulating effects of microglia on neural cell progenitor responses. Co-culturing of microglia treated with LPS (“Mi[LPS]”) blocked both neural progenitor cell (NPC) and OPC differentiation via microglial secretion of pro inflammatory tumor necrosis factor alpha (TNFα) (Butovsky et al., 2006). Furthermore, although both Mi[IFNγ] and Mi[IL-4] co-cultures promoted NPC and OPC differentiation, the effects of Mi[IL-4] on oligodendrogenesis were much more pronounced, potentially through secretion of high levels of insulin-like growth factor 1 (IGF-1) (Butovsky et al., 2006). Our own studies found that microglia treated with IFNγ and LPS (Mi[IFNγ/LPS]) caused expression of iNOS, co-stimulation molecule CD86, and Fc receptor CD16/32 and was associated with *in vitro* induction of OPC proliferation and migration (Miron et al., 2013). Conversely, only treatment with IL-13 (Mi[IL13]) or IL-10 (Mi[IL10]) increased expression of arginase-1, mannose receptor (CD206) and IL1Ra, with conditioned media driving OPC survival under death-inducing conditions and differentiation into mature oligodendrocytes, in part via secretion of activin-A (Miron et al., 2013). Although, these experiments were carried out under artificial environments, they revealed the importance of microglia-derived factors in regulating progenitor responses critical for CNS regeneration.

**Figure 1:**
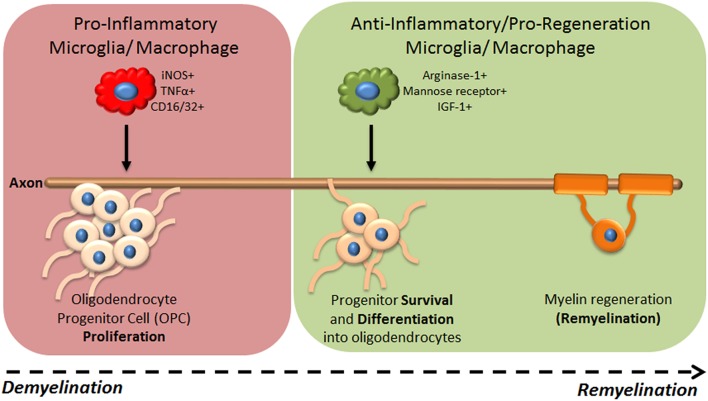
**Microglia/macrophage functional phenotypes during CNS regeneration of myelin**. Following focal demyelination of the adult mouse brain, microglia/macrophages are activated to a pro-inflammatory (iNOS+ TNFα+ CD16/32+) phenotype which drives OPC proliferation but is not required for remyelination to proceed. A switch to an ant-inflammatory/pro-regenerative phenotype (arginase-1+ mannose receptor+ IGF-1+) occurs which is needed for progenitors to differentiate into mature oligodendrocytes and remyelination to subsequently take place.

Recent studies have demonstrated that changes in microglia/macrophage activation regulate their distinct functions during CNS regeneration (summarized in Table 1). For example, in a spinal cord injury model using electromechanical displacement in which axonal regeneration is poor, mRNA expression profiles of genes previously associated with macrophage activation dynamically changed over time post injury (Kigerl et al., 2009). Early time points were associated with increased expression of iNOS, CD16/32, CD86, IFNγ, mannose receptor and arginase-1, whereas expression of mannose receptor and arginase-1 decreased at later time points. This suggested a sustained pro-inflammatory phenotype over the long-term post-injury associated with poor axonal regeneration. This was not rescued by injection of axonal growth-promoting bone marrow derived macrophages (treated *in vitro* with IL-4), as subsequent to injection these cells changed phenotype to mirror the activation phenotype in the lesion. However, direct delivery of IL-4 into the CNS was able to overcome this, and induce a pro-regenerative arginase-1+ macrophage phenotype (Fenn et al., 2014). IL-4 injection also had a pro-regenerative effect in the immune-mediated demyelination mouse model experimental autoimmune encephalitis (EAE), where it increased oligodendrogenesis in the spinal cord, suggesting potential involvement of microglia/macrophages in remyelination. The first definitive evidence of such involvement came from using a lysolecithin (LPC)-induced focal model of demyelination in the mouse spinal cord, showing that depletion of macrophages early after injury using clodronate liposomes lead to a significant delay in remyelination (Kotter et al., 2001). This pro-regenerative role was later associated with phagocytosis of myelin debris, normally inhibitory for remyelination,(Neumann et al., 2009; Lampron et al., 2015) and recruitment of oligodendrocyte progenitor cells (OPCs) to lesions (Kotter et al., 2005). Our group subsequently showed that dynamic temporal regulation of microglia/macrophage activation controls OPC responses during remyelination (Miron et al., 2013; Figure 1). Using LPC-induced focal demyelination in the mouse corpus callosum, we investigated microglia/macrophage activation over time during remyelination using markers previously used in studies of regeneration of the CNS (Kigerl et al., 2009) or of skin and muscle (Deonarine et al., 2007; Ruffell et al., 2009; Dayan et al., 2011). We observed an iNOS+/CD16/32+/TNF-α+ microglia/macrophage population at 3 days post lesion, which switched to an arginase-1+/IGF-1+/mannose receptor+ population at 10 days post lesion. Lineage tracing demonstrated that this switch in activation took place in both microglia and monocyte-derived macrophages. These phenotypic states were associated with distinct functional roles, as depletion of the iNOS+ population using gadolinium chloride (GdCl_3_) (which competitively inhibits calcium signaling and has been previously used to deplete pro-inflammatory macrophages *in vivo*; Hardonk et al., 1992) reduced OPC proliferation, whereas specific depletion of the arginase-1+ population using mannosylated-clodronate liposomes reduced OPC differentiation into mature oligodendrocytes and impaired remyelination (Miron et al., 2013). The pro-remyelination function of this later-arising microglia/macrophage population was demonstrated to be mediated in part by the growth factor activin-A (Miron et al., 2013). In addition, enhanced remyelination in aged animals resulting from parabiotic recruitment of young macrophages to LPC-induced spinal cord lesions (Ruckh et al., 2012) was associated with an increase in arginase-1+ and mannose-receptor+ cells, and not iNOS+ or CD16/32+ microglia/macrophages, thus associating enhanced densities of arginase-1+ macrophages with improved remyelination (Miron et al., 2013). Interestingly, lineage tracing demonstrated that only a small proportion of the arginase-1+ macrophages were derived from the young mouse, showing that old microglia/macrophages adopted the regenerative phenotype when exposed to the young macrophages (Miron et al., 2013). Altogether, these studies have elucidated the dynamic regulation of microglia/macrophages by the CNS microenvironment, resulting in adoption of activation phenotypes that either impede or promote regeneration. A key commonality between these studies is that the temporal regulation of microglia/macrophage activation, i.e., a turning off of “pro-inflammatory” responses and switch to “pro-regenerative” function, is an essential aspect of efficient regeneration.

**Table 1:** **Summary of microglia/macrophage phenotypic and functional characteristics during CNS injury and regeneration *in vivo***.

**Experimental Model**	**Microglia/Macrophage Markers**	**Functional Outcome**	**References**
Spinal cord injury–electromechanical displacement	iNOS, CD16/32, CD86, IFNy, Arginase-1, Mannose Receptor	Poor axonal regeneration associated with decreased Arginase-1 and Mannose Receptor expression	Kigerl et al., 2009
Spinal cord injury–contusion	Arginase-1, IL-1β, IL-4Rα	Poor regeneration associated with lower IL-4Rα expression on monocytes of aged mice	Fenn et al., 2014
		CNS IL-4 injection promoted Arginase-1 in macrophages, increased oligodendrogenesis and improved regeneration	
Experimental Autoimmune Encephalomyelitis (EAE)	iNOS, Arginase-1	Increased iNOS associated with more severe disease	Mikita et al., 2011
		Increased Arginase-1+ macrophages associated with milder disease and recovery of symptoms	
LPC-mediated demyelination of the corpus callosum	3 dpl–iNOS, CD16/32, TNFα	OPC proliferation	Miron et al., 2013
	10 dpl–Arginase-1, Mannose Receptor, IGF-1	OPC differentiation into myelinating oligodendrocytes	
Cuprizone model of demyelination	CX3CR1	CX3CR1 knockout mice displayed insufficient myelin debris clearance and impaired remyelination	Lampron et al., 2015

It has therefore been postulated that an imbalance between the pro-inflammatory and the regenerative macrophage response may contribute to the failure of remyelination in MS. Post-mortem tissue from MS patients has shown an abundance of iNOS+ macrophages in chronic inactive lesions which show poor remyelination, whereas mannose receptor+ macrophages are increased only in actively remyelinating lesions (Miron et al., 2013; Vogel et al., 2013). Interestingly, a population of macrophages with an “intermediate” activation phenotype characterized by expression of CD86, the chemokine CCL22 and costimulatory molecule CD40 whilst being mannose receptor negative predominate in “pre-active” normal appearing white matter and remyelinating lesions, perhaps suggestive of a population of macrophages caught mid-switch (Peferoen et al., 2015). However, an intermediate CD40+ mannose receptor+ phenotype is also abundant in chronic inactive lesions that fail to remyelinate (Vogel et al., 2013) which may represent a pathological block in macrophage phenotypic switching.

The balance between macrophage activation states is predictive of disease severity in EAE. A favoring toward an iNOS+ macrophage population is associated with a significantly higher risk of relapsing EAE, and equilibrium in iNOS+ and arginase-1+ macrophage populations predicts a milder disease (Mikita et al., 2011). In addition, the switch to arginase-1+ macrophages is associated with attenuation of inflammation and decreased disease severity (Ahn et al., 2012). Work from Mikita et al. (2011) was one of the first to recognize the importance of arginase-1+ macrophages in the recovery of EAE and that promotion of the activation of such macrophage populations may represent a novel therapeutic target for MS. Whereas relapse occurrence was predicted by expression of iNOS in circulating monocytes and suppression of arginase-1+ macrophages and microglia, milder pathologies were characterized by low numbers of activated monocytes in the brain stem. Furthermore, therapeutic administration of IL-13- and IL-4-treated monocytes suppressed EAE and decreased disease severity significantly, accompanied by increased arginase-1 expression in the brain stem. These studies suggested that promoting activation of macrophages associated with arginase-1 expression may not only attenuate a pro-inflammatory response, but also promote CNS regeneration.

Overall, recent efforts to investigate the role of microglia/macrophages in CNS regenerative processes, such as remyelination, have identified the importance of temporal control of activation from an initial pro-inflammatory response to a pro-regenerative one. However, the cellular and molecular mechanisms underpinning this switch in activation is unknown but of vital importance in understanding pathological and regenerative mechanisms in MS.

## Plasticity of microglial and macrophage activation during CNS injury and regeneration

Macrophages have long been recognized for their ability to dynamically respond to stimuli in their microenvironment. The concept of “macrophage plasticity” suggests that macrophage activation state is not permanent, and that macrophages retain the ability to adjust their activation in response to additional stimuli in their microenvironment. This is an important concept as it suggests that therapeutically targeting macrophages could switch them from damaging to beneficial/regenerative activation states. Some examples of where this could be applied include aging or in MS, where pro-inflammatory microglia phenotypes have been observed (Miron et al., 2013; Vogel et al., 2013; Grabert et al., 2016). However, whether macrophages have the potential to change their genetic, metabolic and functional signatures to switch to an opposing role under these conditions remains to be determined.

The evidence for the ability of macrophages to undergo a direct phenotypic switch is unclear. *In vitro* studies have attempted to prove that such a switch can occur in macrophages, identified by changes in gene and protein expression. LPS treatment of microglia followed by treatment with IL-4 could not reverse TNF-α production or promote IGF-1 production (Butovsky et al., 2006). Whether this is due to a commitment to an irreversible fate upon pro-inflammatory activation, or to the sheer potency of stimuli like LPS that create robust (and potentially physiologically irrelevant) inflammatory responses remains uncertain. However, other experiments using LPS-treated microglia subsequently treated with IL-4 have shown a significant decrease in the mRNA expression of pro-inflammatory genes iNOS, Cox-2 and CD86, with increased mRNA expression of mannose receptor and Arginase-1 surpassing that observed with IL-4 treatment alone (Chhor et al., 2013). The contradictory evidence for an *in vitro* activation switch may be due to the differences in culture conditions, treatment exposure times and end points. Conversely, it appears that switching macrophages *in vitro* from anti-inflammatory to pro-inflammatory phenotypes may be easier to achieve. IL-4-primed microglia do not produce IL-10 *in vitro*, yet addition of LPS produces a modest IL-10 response indicative of plasticity (Edwards et al., 2006). Furthermore, IL-4-induced IGF-1 production could also be blocked by LPS (Butovsky et al., 2006). The question remains however, whether polarizing macrophages and microglia *in vitro* is physiologically relevant, highlighting the importance of using suitable *ex vivo* or *in vivo* models of tissue microenvironment–specific inflammation to observe the behavior and plasticity of these cells.

There is *in vivo* evidence for changes in macrophage activation following CNS injury. In a model of rodent spinal cord injury through contusion, distinct populations of macrophages are recruited to the lesion site (Kigerl et al., 2009; Shechter et al., 2013). Recruitment is coordinated through two different routes such that Ly6C^hi^CX3CR1^lo^ macrophages associated with expression of IL-1β, TNFα, and IL-12 are recruited through the leptomeninges, and Ly6C^lo^CX3CR1-GFP^hi^ macrophages expressing mannose receptor, IL-10 and TGF-β are recruited via the choroid plexus (Shechter et al., 2013). This research shows how the choroid plexus can prime macrophages to become supportive for repair prior to exposure to the local microenvironment at the lesion site, a previously unidentified mechanism regulating macrophage diversity *in vivo*. Using a focal lysolecithin-induced demyelination model in the corpus callosum, our own studies also demonstrated a change in microglia and monocyte-derived macrophage activation at the onset of remyelination, from iNOS+ TNFα+ CD16/32+ to arginase-1+ mannose receptor+ IGF-1+ phenotypes. These studies demonstrate the diversity of macrophage activation phenotypes following CNS injury and during regeneration; however, the switching in activation of the same population of cells remains to be unequivocally proven.

To gain a greater understanding of potential macrophage plasticity during CNS regeneration (i.e., remyelination), uncovering the cellular and molecular mechanisms that underpin their activation is imperative. Furthermore, identifying key regulators in the balance between the initiation and resolution of inflammatory responses in macrophages may uncover novel therapeutic targets for diseases like MS. Cross-talk between signal transduction pathways involved in both the initiation and resolution of inflammation by macrophages during myelin injury and regeneration results in tightly regulated responses, often by simultaneous activation and inhibition of opposing activation states. For example, msh homeobox 3 (MSX3) promotes pro-repair responses in macrophages that lead to oligodendrocyte survival *in vitro* and suppression of EAE *in vivo*, whilst suppressing pro-inflammatory pathways (Yu et al., 2015). MSX3 leads to activation of critical anti-inflammatory mediators such as PPARγ and STAT6, both of which interact in an inhibitory manner with pro-inflammatory pathways including NFκB (Ohmori and Hamilton, 2000; Remels et al., 2009). Indeed, the NFκB family of transcription factors is among one of the most well characterized pro-inflammatory mediators. Gene expression profiling of LPS-treated microglia *in vitro* have shown that 72 out of 465 NFκB target genes analyzed were significantly repressed or induced (Sharif et al., 2007). Moreover, expression of CD40, a costimulatory molecule associated with autoimmunity and MS, is upregulated in macrophages and microglia following LPS treatment via activation of NFκB (Qin et al., 2005). Its tight autoregulation ensures that its rapid potent activation is controlled and resolved efficiently; however dysregulation can lead to a prolonged inflammatory and neurodegenerative response. Mutations in genes associated with inhibition (Miterski et al., 2002) or constitutive activation of NFκB (De Jager et al., 2009) have been found in MS patients (Housley et al., 2016). Post mortem MS tissue shows strong NFκB activity localized to macrophages near lesions (Gveric et al., 1998), suggesting that a lack of regulation of NFκB may contribute to the inflammation and pathology of the disease. Therefore, blocking key inflammatory pathways may enable a switch in macrophage phenotype and function, leading to the resolution of inflammation and promotion of remyelination.

Other endogenous regulators of inflammation may also provide clues as to how macrophage activation is regulated during CNS injury and remyelination. One example is of PPARγ, whose activation causes its translocation to the nucleus and its binding to NFκB-specific binding sites, preventing NFκB activation and the transcription of pro-inflammatory mediators in a process called trans-repression (Paintlia et al., 2006; Szanto and Nagy, 2008). PPARγ agonist administration *in vivo* can promote functional recovery in a rodent SCI model (McTigue et al., 2007) and neuroprotection when administered as a pre-treatment before EAE induction (Polak et al., 2005), although it is uncertain whether PPARγ agonists can promote recovery in these models through non-macrophage cell types. However, it is known that PPARγ expression is upregulated in microglia treated with IL-4 *in vitro* (Paintlia et al., 2006). *In vitro* PPARγ activation with specific agonists also decreases pro-inflammatory and increases anti-inflammatory marker expression in macrophages infected with the parasite t.Cruzi, a parasitic protozoan which produces a potent pro-inflammatory activation phenotype in macrophages (Penas et al., 2015). PPARγ activation also promotes the transcription of anti-inflammatory molecules like IL-10 (Szanto and Nagy, 2008) and has therefore been identified as a potential activation switch regulator for macrophages. Another example is of substance P, which can promote activation or infiltration of repair-associated macrophages *in vivo* and whose receptor Neurokinin 1 is upregulated in IL-4-treated macrophages (Marriott and Bost, 2000). Substance P administration at the time of spinal cord contusion significantly decreases iNOS, IL-6, and TNF-α mRNA expression whilst increasing IL-10 and Arginase-1 expression. This is associated with increased mannose receptor+ macrophages, conserved myelin sheaths and improved functional recovery (Jiang et al., 2012). An additional example is of 17β-estradiol, the most common estrogen produced by the pre-menopausal ovary, which prevents NFκB activation in macrophages by directly disrupting the microtubules associated with NFκB nuclear translocation, preventing NFκB initiated transcription of pro-inflammatory mediators (Ghisletti et al., 2005) and promoting PPARγ activation (Kumar et al., 2004). 17β-estradiol administration in EAE decreases inflammatory cell infiltration, delays the onset of symptoms and prevents deterioration of neurological function (Feng et al., 2013). This is associated with decreased protein levels of IL-1β, TNF-α, IL-17, Rho kinase II, and increased IL-4 (Feng et al., 2013). *In vitro* treatment of macrophages with 17β-estradiol significantly decreases LPS-induced IL-1α, IL-6, and TNF-α protein expression (Deshpande et al., 1997). In addition, inhibition of an upstream activator of the NFκB pathway, Rho kinase, with Fasudil modulates microglial activation, reduces microglial infiltration to the spinal cord and improves functional outcome in the SOD1 mutant mouse model of amyotrophic lateral sclerosis (ALS) (Tonges et al., 2014). Fasudil also decreases LPS-induced protein expression of TNF-α, IL-6, and inflammatory chemokines CCL3, CCL5 and CXCl1 in microglial cultures *in vitro*.

Whether the dynamics of macrophage activation is regulated by a direct phenotypic switch or recruitment/expansion of different macrophage populations during remyelination is undetermined. Nonetheless, although endogenous inflammation mediators are most likely acting upon a multitude of cell types during CNS repair, it is evident that modulation of key inflammatory pathways can not only alter the activation of microglia and macrophages but can also have significant effects on CNS injury and regeneration in various models of injury. Therefore, finding ways to specifically promote a switch in macrophage activation to pro- regenerative phenotypes represents a novel therapeutic target for diseases like MS.

## Targeting macrophage activity to promote remyelination

Whilst immunomodulatory interventions aiming to dampen initial myelin injury are highly successful in relapse-remitting MS, there are currently no approved therapies to treat the progressive phase of the disease during which failed remyelination and neurodegeneration are prominent. Given the implication of microglia and macrophages in remyelination, global immunosuppression may be detrimental for remyelination in progressive MS. For example, glatiramer acetate, a random polymer of four amino acids of myelin basic protein, is an immunomodulatory drug effective in treating relapse-remitting MS but ineffective in progressive MS. This may be due to its ability to inhibit microglial activity in MS patients (Ratchford et al., 2012).

There are however some drugs in clinical trials specifically aimed at enhancing remyelination. One of these is the anti-LINGO-1 antibody; LINGO-1 is a negative regulator of oligodendrocyte differentiation and myelination, and selective blocking of LINGO-1 promotes remyelination and protects axonal integrity in EAE (Mi et al., 2007). Another promising drug in clinical trials for relapse-remitting and progressive MS is Clemastine, a H1 histamine receptor antagonist. This was identified via high-throughput *in vitro* screening of compounds on cultured OPCs for pro-myelination capacity, as measured by MBP staining surrounding concentric nanopillars (Mei et al., 2014). Clemastine was also found to promote OPC maturation *in vitro* and promote remyelination *in vivo* in the LPC-induced demyelination mouse model (Mei et al., 2014). Clemastine can also modulate macrophage activation and function; for example, it inhibits ATP receptor P2X7-dependant IL-1β release from LPS-primed human macrophages *in vitro* (Norenberg et al., 2011) and increases Arginase-1 and brain derived neurotrophic factor (BDNF) expression levels in macrophages, conferring neuroprotection in the SOD1 mutant mouse model (Apolloni et al., 2016). Whether the Clemastine can promote remyelination *in vivo* via modulation of macrophage activation remains to be determined.

As of yet there are no MS therapies aimed specifically at modulating microglial/macrophage function to promote remyelination, although an experimental study emerged recently which therapeutically targeted microglia and macrophages to promote remyelination. Using a combination of the antifungal drug Amphotericin B and macrophage colony stimulating factor (M-CSF) to treat mice with LPC-induced demyelinated lesions in the spinal cord caused a significant increase in macrophage and microglia infiltration to the lesioned areas accompanied by an increase in the scavenger marker MSR-1 on CD68+ activated macrophages and microglia, indicative of early myelin debris clearance. This was associated with increased numbers of OPCs and a higher proportion of remyelinated axons (Doring et al., 2015), effects abrogated by clondronate liposome-mediated depletion of peripheral monocytes.

Although, promising, the high toxicity and extensive side effects associated with amphotericin B suggest its translation to a mainstay prolonged treatment in MS is unlikely. Together with the unproven efficacy of anti-LINGO-1 antibody and Clemastine, this highlights the need for development of novel strategies to promote remyelination in MS. Whereas microglia and macrophages have been targeted experimentally by immunomodulatory drugs, Estradiol, NFκB modulators or PPARγ agonists, potential effects on other cell types may render therapeutic application difficult. Therefore, developing specifically targeted therapeutics which effectively and specifically modulate macrophage activation to promote remyelination would be ideal in minimizing off-target interactions and unwanted side effects.

## Conclusion

The functional diversity of CNS macrophages reflects their ability to acquire tissue microenvironment-specific activation phenotypes. During efficient CNS regeneration, such as remyelination, such diversity is observed as a temporally controlled switch in activation from pro-inflammatory to pro-regenerative. However, the cellular and molecular mechanisms underpinning this plasticity of macrophage activation remain to be uncovered. This is critical for the development of novel pro-remyelination strategies for MS that would center on the manipulation of macrophage activation toward phenotypes that are involved in inflammation resolution and are supportive of remyelination.

## Author contributions

All authors listed, have made substantial, direct and intellectual contribution to the work, and approved it for publication.

## Funding

AL is funded by an Industrial CASE studentship from BBSRC and GlaxoSmithKline. VM is supported by a career development award from the UK MS Society and the Medical Research Council.

### Conflict of interest statement

VM has funding and collaborations from Biogen Idec, Glaxo Smith Kline, the Centre for Drug Research and Development and BVBioMed, and has in the past five years performed consultancy for Novartis. The authors declare that the research was conducted in the absence of any commercial or financial relationships that could be construed as a potential conflict of interest.
